# Concurrent training and interindividual response in women with a high number of metabolic syndrome risk factors

**DOI:** 10.3389/fphys.2022.934038

**Published:** 2022-09-23

**Authors:** Pedro Delgado-Floody, Luis Chirosa-Ríos, Felipe Caamaño-Navarrete, Pablo Valdés-Badilla, Tomás Herrera-Valenzuela, Matías Monsalves-Álvarez, Cristian Núñez-Espinosa, Mauricio Castro-Sepulveda, Eduardo Guzmán-Muñoz, David C. Andrade, Cristian Álvarez

**Affiliations:** ^1^ Department of Physical Education, Sport and Recreation, Universidad de La Frontera, Temuco, Chile; ^2^ Department Physical Education and Sports, Faculty of Sport Sciences, University of Granada, Granada, Spain; ^3^ Physical Education Career, Universidad Autónoma de Chile, Temuco, Chile; ^4^ Department of Physical Activity Sciences, Faculty of Education Sciences, Universidad Católica del Maule, Talca, Chile; ^5^ Carrera de Entrenador Deportivo, Escuela de Educación, Universidad Viña del Mar, Viña del Mar, Chile; ^6^ Escuela de Ciencias de la Actividad Física, el Deporte y la Salud, Universidad de Santiago de Chile (USACH), Santiago, Chile; ^7^ Instituto de Ciencias de la Salud, Universidad de O’Higgins, Rancagua, Chile; ^8^ Human Performance Laboratory, Motion Training, Rehab and Nutrition, Lo Barnechea, Chile; ^9^ School of Medicine, University of Magallanes, Punta Arenas, Chile; ^10^ Centro Asistencial de Docencia e Investigación, Punta Arenas, Chile; ^11^ Interuniversity Center for Healthy Aging, Chile, Chile; ^12^ Laboratorio de Fisiología del Ejercicio y Metabolismo (LABFEM), Escuela de Kinesiología, Facultad de Medicina, Universidad Finis Terrae, Santiago, Chile; ^13^ School of Kinesiology, Faculty of Health, Universidad Santo Tomás, Santiago, Chile; ^14^ Exercise Applied Physiology Laboratory, Centro de Investigación en Fisiología y Medicina de Altura, Departamento Biomédico, Facultad de Ciencias de la Salud, Universidad de Antofagasta, Antofagasta, Chile; ^15^ Exercise and Rehabilitation Sciences Institute, School of Physical Therapy, Faculty of Rehabilitation Sciences, Universidad Andres Bello, Santiago, Chile

**Keywords:** morbid obesity, physical activity, exercise, sleep quality, quality of life

## Abstract

The non-responders (NRs) after exercise training have been poorly studied in populations with morbid obesity. The purpose of this study was to determine the NR prevalence after 20 weeks of concurrent training of morbidly obese women with a high or low number of metabolic syndrome (MetS) risk factors. Twenty-eight women with morbid obesity participated in an exercise training intervention and were allocated into two groups distributed based on a high (≥3, *n* = 11) or low number (<3, *n* = 17) of MetS risk factors. The main outcomes were waist circumference (WC), fasting plasma glucose (FPG), high-density lipids (HDL-c), triglycerides (Tg), and systolic (SBP) and diastolic (DBP) blood pressure, and secondary outcomes were body composition, anthropometric and physical fitness, determined before and after 20 weeks of concurrent training. NRs were defined as previously used technical error cut-off points for the MetS outcomes. Significantly different (all *p* < 0.05) prevalences of NRs between the H-MetS vs. L-MetS groups (respectively) in WC (NRs 18.2 % vs. 41.1 %, *p* < 0.0001), SBP (NRs 72.7 % vs. 47.0 %, *p* = 0.022), DBP (NRs 54.5 % vs. 76.4 %, *p* < 0.0001), FPG (NRs 100% vs. 64.8 %, *p* < 0.0001), and HDL-c (NRs 90.9 % vs. 64.7 %, *p* = 0.012) were observed. In addition, the H-MetS group evidenced significant changes on ΔSBP (−10.2 ± 11.4 mmHg), ΔFPG (−5.8 ± 8.2 mg/dl), ΔHDL-c (+4.0 ± 5.9 mg/dl), and ΔTg (−8.8 ± 33.8 mg/dl), all *p* < 0.05. The L-MetS group only showed significant changes in ΔWC (−3.8 ± 5.0 cm, *p* = 0.009). Comparing H-MetS vs. L-MetS groups, significant differences were observed in ∆FPG (−5.8 ± 8.2 vs. +0.3 ± 3.2 mg/dl, *p* = 0.027), but not in other MetS outcomes. In conclusion, 20 weeks of concurrent training promotes greater beneficial effects in morbidly obese patients with a high number of MetS risk factors. However, the NR prevalence for improving MetS outcomes was significantly superior in these more-diseased groups in SBP, FPG, and HDL-c, independent of their major training-induced effects.

## Introduction

Severe obesity is a chronic disease with abnormal or excessive fat accumulation that presents a health risk, substantially increasing the rates of total mortality, but with an important reduction in life expectancy in comparison with normal-weight peers (Kitahara et al., 2014). Despite the weight and fat accumulation, candidates for bariatric surgery suffer from several other cardiometabolic consequences, such as metabolic syndrome (MetS) (Baffi et al., 2016). Moreover, severe obesity has been associated with impairments of fitness, limiting the capacity to perform activities of daily life (Pazzianotto-Forti et al., 2020). Therefore, managing MetS risk factors plays a fundamental role in severely obese women (Alberti et al., 2009). Additionally, the percentage of women who have morbid obesity is higher than that of their male peers who suffer from this disease (Basterra-Gortari et al., 2011). The evidence projects that by 2030, morbid obesity will be the most common disease category among women in the US (Ward et al., 2019).

Exercise training and particularly, concurrent training i.e., a mixture of a high-intensity interval [i.e., HIIT, a few seconds of high-intensity intervals on a bike interspersed by recovery periods (Gibala et al., 2012)] plus resistance training (i.e., RT, voluntary concentric/eccentric muscle contraction using external weights) (Dunstan et al., 2002) is not only a useful tool for counteracting altered anthropometric/body composition, cardiovascular, and metabolic parameters with health aims (Delgado-Floody et al., 2021; Dupuit et al., 2021) but is also a feasible strategy for improving the overall physical performance and mental condition in patients with morbid obesity (Delgado-Floody et al., 2020; Delgado-Floody et al., 2021). Of note, concurrent training intervention can change body weight, body fat, waist circumference (WC), and systolic and diastolic blood pressure in morbidly obese patients (Picó-Sirvent et al., 2019). We have corroborated these findings from our own experience in these cohorts with MetS risk factors (Delgado-Floody et al., 2020; Delgado-Floody et al., 2021).

Interestingly, it has been demonstrated that not all subjects develop similar adaptations to the same exercise training program, which is called interindividual variability to exercise training (Bouchard et al., 2012; Álvarez et al., 2019). Indeed, some subjects may achieve benefits, thus being responders (Rs), whereas others may show a worsened or unchanged response, known as non-responders (NRs) (Bouchard et al., 2012; Bonafiglia et al., 2016). There has been a wide range of NR prevalence to a determined outcome reported where several “factors”, such as exercise modality, sex, biological maturation, and baseline conditions (anthropometrics and fitness) (Davidsen et al., 2011), which could play a fundamental role in exercise–response, have been identified.

Of note, although R and NR phenotypes have been identified in several populations, there is scarce evidence related to R and NR prevalence on the MetS risk factors in morbidly obese patients after concurrent training intervention (Álvarez et al., 2018). As previously reported, the effects of exercise training in patients with more altered baseline conditions usually not only decrease the NR prevalence (i.e., almost all participants improve their condition) (Álvarez et al., 2017) but also, the literature is clear that being an NR to a particular outcome is not mandatory to be an NR to another outcome after exercise training (Astorino and Schubert, 2014). Thus, the objective was to determine the NR prevalence after 20 weeks of concurrent training on morbidly obese women with a high or a low number of MetS risk factors. Accordingly, we hypothesized that concurrent training promotes similar beneficial changes in both groups, with a high and a low number of MetS risk factors, and that NR prevalence (i.e., % of NRs) could not always be represented by the H-MetS group that characterizes those subjects with major disease states as regularly as the literature has shown.

## Materials and methods

### Study design and participants

This was a quasi-experimental study developed for severely obese women of the Morbid Obesity Association for Bariatric Surgery Candidates of Temuco, Chile. The patients were invited to participate in a public meeting, and after all information and feedback about the risk/benefits were shared, all participants signed informed consent.

The inclusion criteria were as follows: 1) age >18 and <60 years, 2) female, 3) medical authorization for physical tests, and 4) the body mass index (BMI) equal to or greater than 30.0 with obesity-related health conditions. The exclusion criteria were as follows: 1) physical limitations to performing the physical test (e.g., restrictive injuries of the musculoskeletal system), 2) exercise-related dyspnea or respiratory alterations, and 3) chronic heart disease with any degree of worsening in the last month.

In the enrollment stage, 46 (*n* = 46) participants with two or more risk factors for MetS showed their intention to participate. However, after screening of inclusion/exclusion criteria, only 37 (*n* = 37) were recruited. From here, participants were allocated according to the number of MetS risk factors: either a high number of MetS risk factors ≥3 (H-MetS, *n* = 15) or a low number of MetS risk factor group <3 (L-MetS, *n* = 22). After 20 weeks of follow-up, nine (*n* = 9) participants were excluded for several reasons (*n* = 4 excluded from the H-MetS group, and *n* = 5 excluded from the L-MetS group). Thus, the final sample size was *n* = 28 (H-MetS, *n* = 11 and L-MetS, *n* = 17). The sample size was previously calculated using the G*Power, version 3.1.2. Delta changes from SBP, a standard deviation (6.0 mmHg), and a critical *t* value of 1.73 from previous similar studies in exercise training (Delgado-Floody et al., 2019) yielded a total sample size of *n* = 10 participants per group. The study was carried out following the Declaration of Helsinki (2013) and was approved by the Ethical Committee of the Universidad de La Frontera, Temuco, Chile. A flow diagram of the study participants can be seen in (Supplementary Material S1).

### Metabolic syndrome risk factor measurement

The MetS outcomes were screened using standard criteria (Alberti et al., 2009). After overnight fasting for 10 ± 2 h, all patients underwent a baseline assessment (pre-test) between 08:00 and 9:00 in the morning, where they arrived at the laboratory (health center) for blood sample extraction of 5 ml to determine the MetS outcomes: fasting plasma glucose (FPG), high-density lipoprotein cholesterol (HDL-c), and triglycerides (Tg). Additional metabolic outcomes such as the total cholesterol (Tc) and low-density lipids (LDL-c) were also included. SBP and DBP were measured according to the standard criteria (Mancia et al., 2014). Blood pressure was measured with an OMRON™ digital electronic BP monitor (model HEM 7114, Chicago, IL, United States) in a sitting position after a 5-min rest period. Two recordings were made, and the mean of both measurements was used for statistical analysis. Patients were advised to avoid caffeine, exercise, and smoking for at least 30 min before measurement (Chobanian et al., 2003). The WC was assessed with an inextensible tape measure graduated in centimeters (Adult SECA™) at the upper hipbone and the top of the right iliac crest, with an elastic measuring tape in a horizontal plane around the abdomen at the level of the iliac crest. The tape was snug, but it did not compress the skin and was parallel to the floor. The measurement was made at the end of a normal expiration (North American Association for the Study of Obesity et al., 2000).

### Responders and non-responders to metabolic syndrome risk factors

Following the previous original authors (Bouchard et al., 2012), the interindividual variability to exercise training was reported as responders (Rs) and non-responders (NRs), using the typical error measurement (TE). Thus, using two previous measurements of our participants in these five MetS compounds of the MetS, we calculated the TE. Then, we used the TE × 2 calculated for WC (0.50 cm × 2 cm = 1.0 cm), SBP (4.01 mmHg × 2 mmHg = 8.02 mmHg), DBP (2.49 mmHg × 2 mmHg = 4.98 mmHg), HDL-c (2.5 mg/dL × 2 mg/dL = 5.0 mg/dL), triglycerides (12.3 mg/dL × 2 mg/dL = 24.6 mg/dL), and FPG (3.5 mg/dL × 2 mg/dL = 7.0 mg/dL) using the known equation: TE = *SD*_*diff*_/2, where *SD*_diff_ is the variance (standard deviation) of the different scores observed between the two repetitions of each test. The NRs for all the MetS outcomes were defined as those individuals who failed to demonstrate a decrease or increase (in favor of beneficial changes) that was greater than twice the TE away from zero.

### Body composition measurement

Body fat (%), lean mass (kg), and skeletal muscle mass (kg) were measured using a digital bioimpedance scale (TANITA™, model 331, Tokyo, Japan). Body mass was measured with the TANITA™ model 331 (Tokyo, Japan) and height (m) with a SECA™ stadiometer (model 214, Hamburg, Germany). On the day of the measurement, subjects wore light clothing and were without shoes. The BMI was calculated as the bodyweight divided by the square of the height (kg/m^2^). The BMI was determined to estimate the degree of obesity (kg/m^2^) using the standard criteria for obesity and severe obesity classification (Sturm, 2007; Johnson-Stoklossa et al., 2017).

### Endurance performance

The physical condition of the participants in both H-MetS and L-MetS groups was measured by endurance and muscle strength testing. First, a 6-min walking test was used to estimate cardiorespiratory fitness. The test was performed in a closed space on a flat surface (30 m long), with two reflective cones placed at the ends to indicate the distance. During the test, participants were assisted with instructions from an exercise physiologist (De Souza et al., 2009).

### Muscle strength

Handgrip strength was assessed using a digital dynamometer (Baseline™ Hydraulic Hand Dynamometers, United States), which has been used in previous studies (Norman et al., 2011). Two attempts were made, measuring each hand, and the best result from each was selected. As mentioned previously, the mean value obtained was taken as the final score (Norman et al., 2011).

### Quality of life determination

Health-related quality of life outcomes (HRQoL) were measured according to the SF-36 questionnaire (Karlsen et al., 2011). This instrument measured eight domains related to physical and mental health: 1) physical functioning, 2) role limitations due to physical problems and physical role, 3) bodily pain, 4) general health perceptions, 5) vitality, 6) social functioning, 7) role limitations due to emotional problems, and 8) emotional role and emotional well-being. The first four domains constitute the summary of the physical health component, and the last four domains constitute the summary of the mental health component. The scores on all the domains are transformed into a scale from 0 to 100, where the highest score indicates the optimal and the lowest the poorest HRQoL score. In this study, we reported the physical and mental dimensions’ summary (0–100 points) and the overall score of HRQoL (0–100 points).

### Intervention

The concurrent training program had two sections of RT and HIIT, which were applied at a frequency of 2 days per week. Before the start of the exercise program, each participant was involved in four familiarization sessions that included the following: 1) knowledge of all measurements, exercise machines, and instructions during the exercise program; 2) exercising in cycling, weights, and metal bars; and 3) applying a few exercises of HIIT by two to three intervals and RT in two to three sets of exercises to know the configuration of each exercise of their concurrent training program. Following this, in the first RT section, three out of four RT exercises were included by each participant (according to the planning week), targeting the following muscle groups: 1) forearm, 2) knee flexors and extensors, 3) trunk, 4) chest, 5) shoulder elevators, 6) horizontal shoulder flexors, 7) extensors, and finally, 8) plantar flexors. These exercises were performed in three sets of as many repetitions (continuous concentric/eccentric voluntary contraction) as possible in 60 s, followed by 60–120 s of passive recovery. The exercise intensity was identified with a load that promotes muscle failure (i.e., 8–10 points of the modified subjective perception Borg scale intensity), which in terms of one-maximum repetition test was in a range between 20 % and <50% of the 1RM. About the recovery period, from 120 s in the first week, participants progressively decreased this time until finishing with only 60 s of recovery between sets, as previously reported (Álvarez et al., 2019). The HIIT section consisted of 60 s of a maximum-intensity exercise using a magnetic resistance static bicycle (Oxford™ Fitness, model BE-2701, Chile), followed by 60–120 s of passive recovery over the bicycle without movement. This was repeated four to seven times according to the weekly schedule (Delgado-Floody et al., 2019). The exercise intensity was measured on the Borg scale of 1–10 of perceived exertion, and the participants worked at a level between 6 to 9 points. All sessions started with a 10-min warm-up period with continuous walking and joint mobility and flexibility exercises, followed by 5–10 min of cool down and stretching to prevent injuries. Each concurrent training session had a time duration of 60 min/session, accumulating to 120 min/week.

### Statistical analyses

Data are presented as the mean and standard deviation in the tables and interindividual results to report the interindividual variability to exercise training. The normality distribution for all data was analyzed using the Shapiro–Wilk test. For training-induced changes from pre to-post test, a repeated-measure two-way ANOVA (group x 2 times) was applied to assess the occurrence of an actual training effect; namely, *p* < 0.05 for the interaction [time (2; pre and post) × group (2; H-MetS and L-MetS group)] on the main MetS markers (WC, SBP, DBP, FPG, HDL-c, and Tg, as well as to the secondary anthropometrics, body composition, metabolism, endurance, and strength performance outcomes). After that, the delta (*Δ*) changes in each main and secondary outcome were calculated, where Student’s *t*-test was used to compare the differences in delta adaptations between the H-MetS vs. the L-MetS group. The comparisons of *∆*s in the outcomes (FPG, Tg, mental dimension score) were analyzed using the unpaired *t*-test and non-parametric results with the Mann–Whitney non-parametric test. The Cohen *d* effect size was obtained with threshold values at 0.20, 0.60, 1.2, and 2.0 for “*small*,” “*moderate*,” “*large*,” and “*very large*” effect sizes, respectively (Hopkins et al., 2009). These procedures were applied using the statistical software Graph Pad Prism, version 8.0. Additionally, the prevalence of NRs was described in percentages (%) in each H-MetS and L-MetS group, and the chi-square test χ^2^ test was applied for comparing the NR prevalence among the frequencies of the groups. This procedure was performed using SPSS statistical software, version 28.0 (SPSS™ Inc., Chicago, IL, United States). The alpha level was set at *p* < 0.05 for statistical significance.

## Results

### Training-induced changes in the main metabolic syndrome outcomes

**FIGURE 1:**
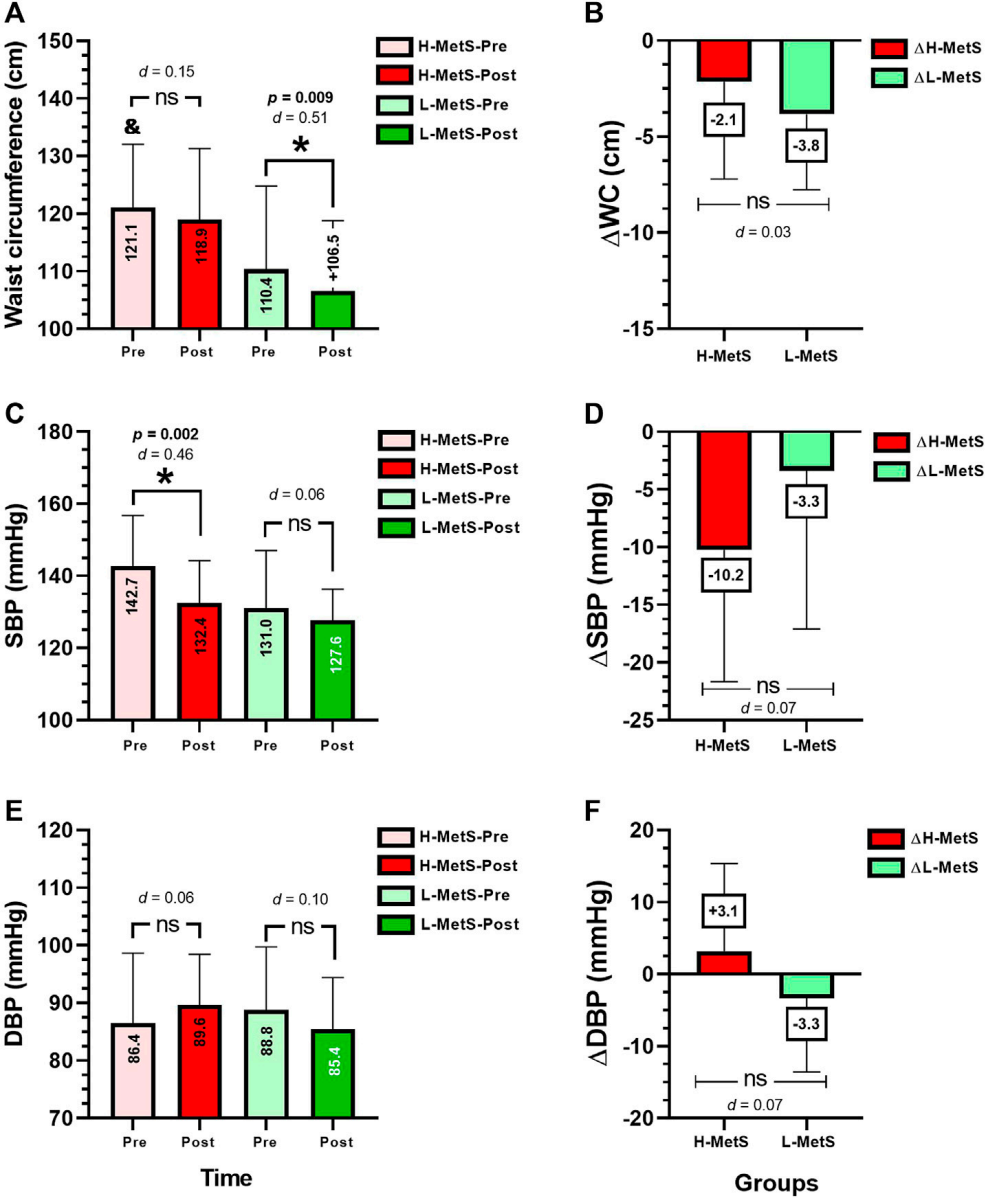
Metabolic syndrome outcomes before and after **(A,C,E)**, and delta changes **(B,D,F)** between two groups of a different number of risk factors for metabolic syndrome. Groups are described as H-MetS-Pre, high number of metabolic syndrome risk factors groups at pre-test; H-MetS-Post, high number of metabolic syndrome risk factors groups at post-test; L-MetS-Pre, low number of metabolic syndrome risk factors group at pre test; L-MetS-Post, low number of metabolic syndrome risk factors group at post test. Outcomes are described as; WC, waist circumference; SBP, systolic blood pressure; DBP, diastolic blood pressure. (&) Denotes the significantly different versus baseline L-MetS group at *p* < 0.05. (*d*) Denotes Cohen *d* effect size at *p* < 0.05. (*) Denotes the significant differences between the H-MetS vs. L-MetS group at *p* < 0.05. (ns) Denotes no significant differences between groups.

After the intervention, there were significant changes in the L-MetS group in absolute values of WC from pre to post test (110.4 ± 14.2–106.5 ± 12.2 cm, *p* = 0.009) (Figure 1A). The H-MetS group showed significant changes in SBP (142.7 ± 14.0–132.4 ± 11.7 mmHg, *p* = 0.002) (Figure 1C). Comparing delta (*Δ*) changes between the H-MetS vs. L-MetS group, no significant differences were observed in the delta values of ΔWC, ΔSBP, and ΔDBP (Figures 1B,D,F).

**FIGURE 2:**
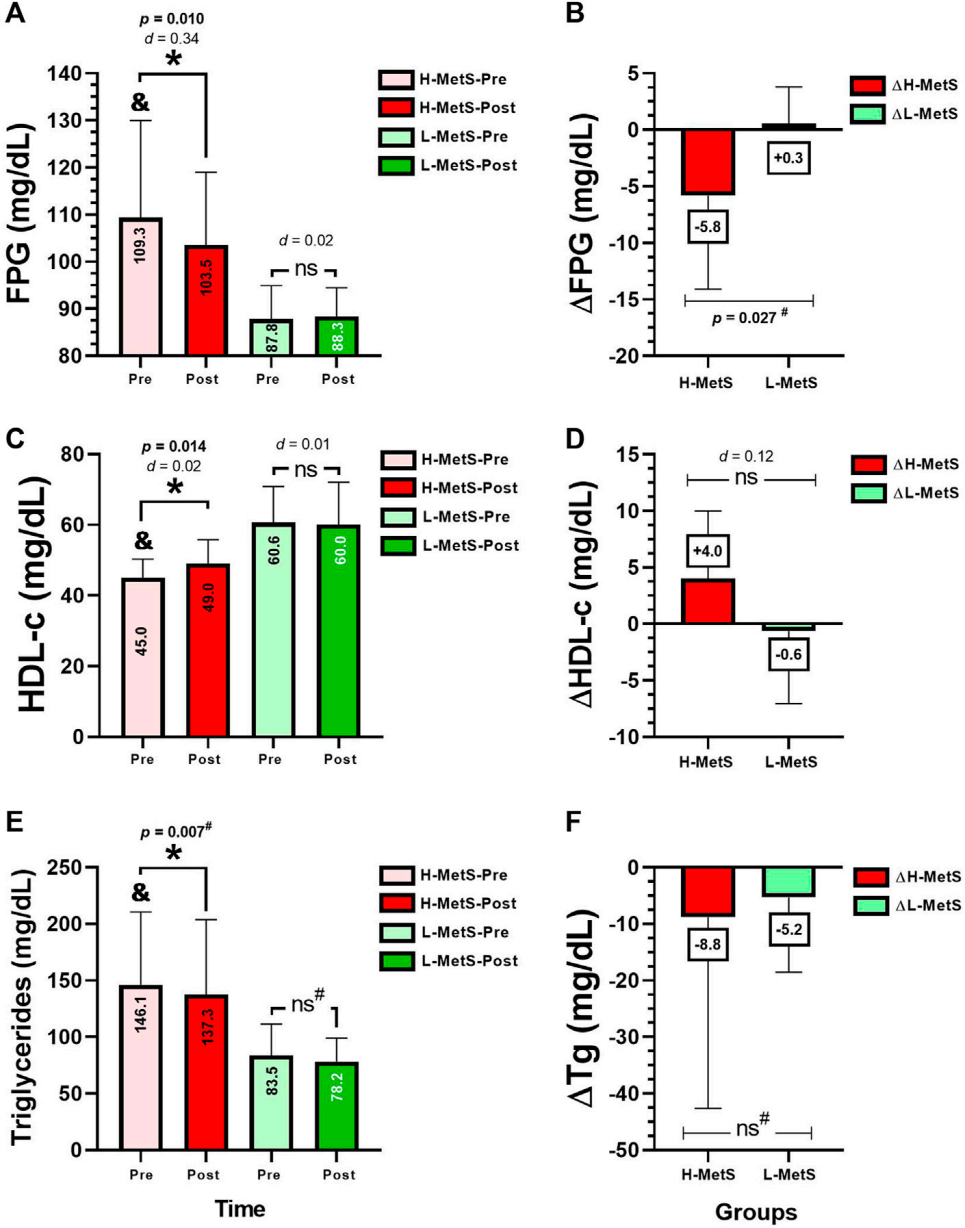
Metabolic syndrome outcomes before and after **(A,C,E)**, and delta changes **(B,D,F)** between two groups of a different number of risk factors for metabolic syndrome. Groups are described as H-MetS-Pre, high number of metabolic syndrome risk factors group at pre-test; H-MetS-Post, high number of metabolic syndrome risk factors group at post-test; L-MetS-Pre, low number of metabolic syndrome risk factors group at pre-test; L-MetS-Post, low number of metabolic syndrome risk factors group at post-test. Outcomes are described as; FPG, fasting plasma glucose; HDL-c, high-density lipoprotein cholesterol; Tg, triglycerides. (&) Denotes significantly different versus baseline L-MetS group at *p*<0.05. (*d*) Denotes Cohen *d* effect size at *p* < 0.05. (*) denotes significant differences between the H-MetS vs*.* L-MetS group at *p* < 0.05. (ns) denotes no significant differences between groups. (^#^) Denotes the comparison analyzed by the Mann–Whitney non-parametric test.

After the intervention, there were significant changes in the H-MetS group in the absolute values of FPG from pre to post test (109.3 ± 20.6–103.5 ± 15.4 mg/dL, *p* = 0.010, *d* = 0.34) (Figure 2A). There were significant changes in the H-MetS group in HDL-c from pre to post test (45.0 ± 5.3–49.0 ± 6.7 mg/dL, *p* = 0.014, *d* = 0.02) (Figure 2C). There were significant changes in the H-MetS group in Tg from pre to post test (146.1 ± 64.4–137.3 ± 66.1 mg/dL, *p* = 0.007, *d* = 0.02) (Figure 2E). Comparing the delta changes between H-MetS vs. L-MetS groups, significant differences were observed in delta changes in ∆FPG (−5.8 vs. +0.3 mg/dL, *p* = 0.027) (Figure 2B).

### Interindividual response for improving metabolic syndrome outcomes

**FIGURE 3:**
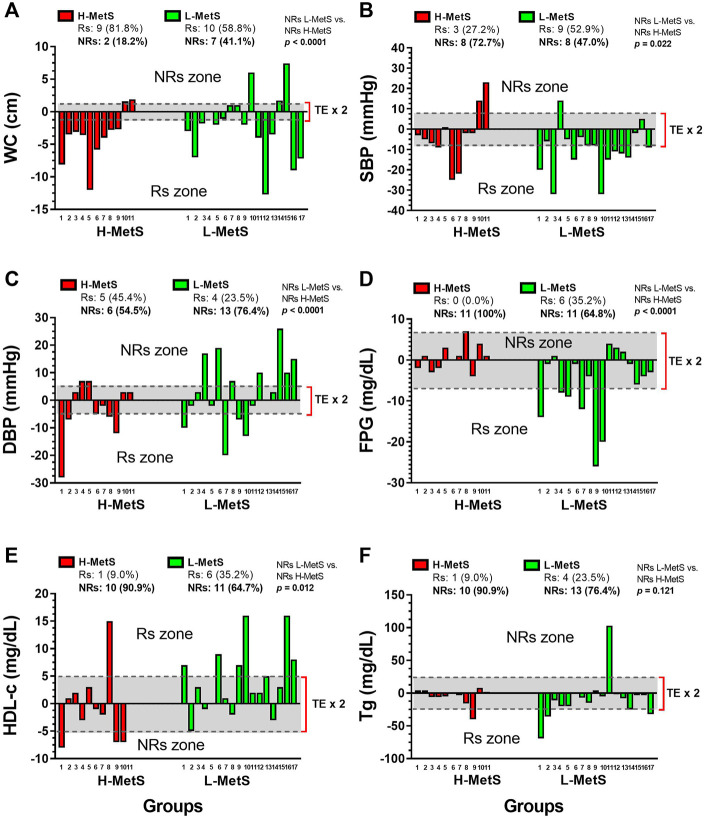
Interindividual changes in metabolic syndrome markers between two groups of a different number of risk factors for the metabolic syndrome. Groups are described as H-MetS, high number of metabolic syndrome risk factors group; L-MetS, low number of metabolic syndrome risk factors group; and L-MetS, low number of metabolic syndrome risk factors group at post test. Outcomes are described as WC (panel **A**), waist circumference; SBP, systolic blood pressure (panel **B**); DBP, diastolic blood pressure (panel **C**); FPG, fasting plasma glucose (panel **D**); HDL-c, high-density lipoprotein cholesterol (panel **E**); Tg, triglycerides (panel **F**); Rs, responders; and (NRs) non-responders for improving MetS outcomes. (NRs zone) Denotes all the gray areas in which participants classified as NRs using the TE parameter. TE, technical error of measurement.

We tested the Rs and NRs for improving WC, SBP, DBP, FPG, HDL-c, and Tg in both H-MetS and L-MetS groups. There were significant differences in the prevalence of NRs between the H-MetS vs. L-MetS groups in the outcomes WC (NRs 18.2 % vs. 41.1 %, *p* < 0.0001) (Figure 3A), SBP (NRs 72.7 % vs. 47.0 %, *p* = 0.022) (Figure 3B), DBP (NRs 54.5 % vs. 76.4 %, *p* < 0.0001) (Figure 3C), FPG (NRs 100 % vs. 64.8 %, *p* < 0.0001) (Figure 3D), and HDL-c (NRs 90.9 % vs. 64.7 %, *p* = 0.012) (Figure 3E).

### Training-induced changes in secondary outcomes

At the baseline, there were significant differences comparing the H-MetS vs. L-MetS groups in secondary outcomes BMI (45.5 ± 6.0 vs. 38.3 ± 6.2, *p* = 0.007), body fat (49.9 ± 3.3 vs. 45.5 ± 5.6 kg, *p* = 0.015), and lean mass (58.0 ± 7.7 vs. 50.1 ± 5.3 kg, *p* = 0.006) (Table 1).

**TABLE 1:** Characteristics of the morbidly obesity with high and low loads of metabolic syndrome risk factors after 20 weeks of patient participants of two concurrent training.

	Time	H-MetS	L-MetS	Baseline *p* value^†^	∆H-MetS vs. ∆L-MetS, *p* value, *η* ^2^ *=*
(*n* = )		11	17		
Age (y)		44.2 ± 11.4	37.5 ± 11.5	*p* = 0.141	
Anthropometric					
Body mass (kg)	Pre	115.4 ± 17.8	93.6 ± 17.7	*p* = 0.726	*p* = 0.107^#^
	Post	112.6 ± 16.9^††^	92.3 ± 17.1		
	∆_kg_	−2.7 ± 0.9	−1.3 ± 0.6		
	∆_%_	−2.4	−1.3		
BMI (kg/m^2^)	Pre	45.5 ± 6.0	38.3 ± 6.2	***p* = 0.007**	*p* = 0.149^#^
	Post	44.5 ± 6.0^††^	37.8 ± 5.8		
	∆_kg/m2_	−1.1 ± 0.1	−0.5 ± 0.4		
	∆_%_	−2.1	−1.3		
Body composition					
Body fat (kg)	Pre	49.9 ± 3.3	45.5 ± 5.6	***p* = 0.015**	*p* = 0.924^#^
	Post	49.7 ± 3.4	45.3 ± 4.7		
	∆_kg_	−0.2 ± 0.1	−0.2 ± 0.9		
	∆_%_	−0.4	−0.4		
Lean mass (kg)	Pre	58.0 ± 7.7	50.1 ± 5.3	***p* = 0.006**	*p* = 0.334, *η* ^2^ = 0.12
	Post	56.8 ± 7.4^†^	50.6 ± 5.8		
	∆_kg_	−1.2 ± 0.3	+0.5 ± 0.5		
	∆_%_	−2.0	+0.9		
Skeletal muscle mass (kg)	Pre	55.1 ± 7.3	47.6 ± 5.0	*p* = 0.063	*p* = 0.142, *η* ^2^ = 0.08
	Post	53.7 ± 7.3^†^	47.5 ± 5.5		
	∆_kg_	−1.3 ± 0.1	−0.1 ± 0.5		
	∆_%_	–2.5	–0.2		
Metabolic					
Total cholesterol (mg/dL)	Pre	181.7 ± 33.1	180.2 ± 14.0	*p* = 0.886	*p* = 0.783, *η* ^2^ = 0.02
	Post	170.9 ± 34.7^†^	171.5 ± 21.0		
	∆_mg/dL_	−10.7 ± 1.6	−8.6 ± 7.0		
	∆_%_	−5.9	−4.8		
LDL-c (mg/dL)	Pre	116.4 ± 37.0	117.0 ± 16.4	*p* = 0.409	*p* = 0.211, *η* ^2^ = 0.05
	Post	120.6 ± 24.9	113.0 ± 18.9		
	∆_mg/dL_	+4.2 ± 12.1	−4.0 ± 2.5		
	∆_%_	−3.6	−3.4		
Endurance performance					
Six-minute walking test (m)	Pre	516.8 ± 95.9	520.9 ± 108.2	*p* = 0.414	*p* = 0.472, *η* ^2^ = 0.02
	Post	615.9 ± 114.5^†^	571.8 ± 224.0		
	∆_m_	+99.1 ± 18.6	+50.9 ± 115.8		
	∆_%_	+19.1	+9.7		
Strength performance					
Handgrip strength (kg)	Pre	29.5 ± 9.2	28.7 ± 7.7	*p* = 0.800	*p* = 0.851, *η* ^2^ = 0.001
	Post	31.8 ± 9.4^†^	30.2 ± 8.6		
	∆_kg_	+2.4 ± 0.2	+1.3 ± 0.9		
	∆_%_	+7.7	+5.2		
Sleep quality (score)	Pre	6.4 ± 3.8	4.9 ± 3.4	*p* = 0.301	*p* = 0.282, *η* ^2^ = 0.04
	Post	3.9 ± 2.8^†^	3.9 ± 2.6		
	∆_pts_	–2.4 ± 1.0	–1.0 ± 0.8		
	∆_%_	–39.0	–20.4		

Data are shown as mean and ±SD. Outcomes are described as BMI, body mass index; LDL-c, low-density lipid cholesterol. (^†^) Denotes *p* < 0.05. (^††^) Denotes *p* < 0.001. Within-group analyses were applied by 2-way ANOVA group x time. Significant differences are in bold.

In the H-MetS group, the within-group comparisons from pre to post test revealed significant changes in the weight (115.4 ± 17.8–112.6 ± 16.9 kg, *p* < 0.001), BMI (45.5 ± 6.0–44.5 ± 6.0 kg/m^2^, *p* < 0.001), lean mass (58.0 ± 7.7–56.8 ± 7.4 kg, *p* = 0.003), skeletal muscle mass (55.1 ± 7.3–53.7 ± 7.3 kg, *p* = 0.015), total cholesterol (181.7 ± 33.1–170.9 ± 34.7 mg/dL, *p* = 0.034), 6-min walking test (516.8 ± 95.9–615.9 ± 114.5 m, *p* = 0.011), HGS (29.5 ± 9.2–31.8 ± 9.4 kg, *p* = 0.017), and sleep quality (6.4 ± 3.8–3.9 ± 2.8 pts., *p* = 0.007) (Table 1). Comparing the H-MetS vs. L-MetS groups at delta changes, no significant differences between the groups were detected, (Table 1).

**FIGURE 4:**
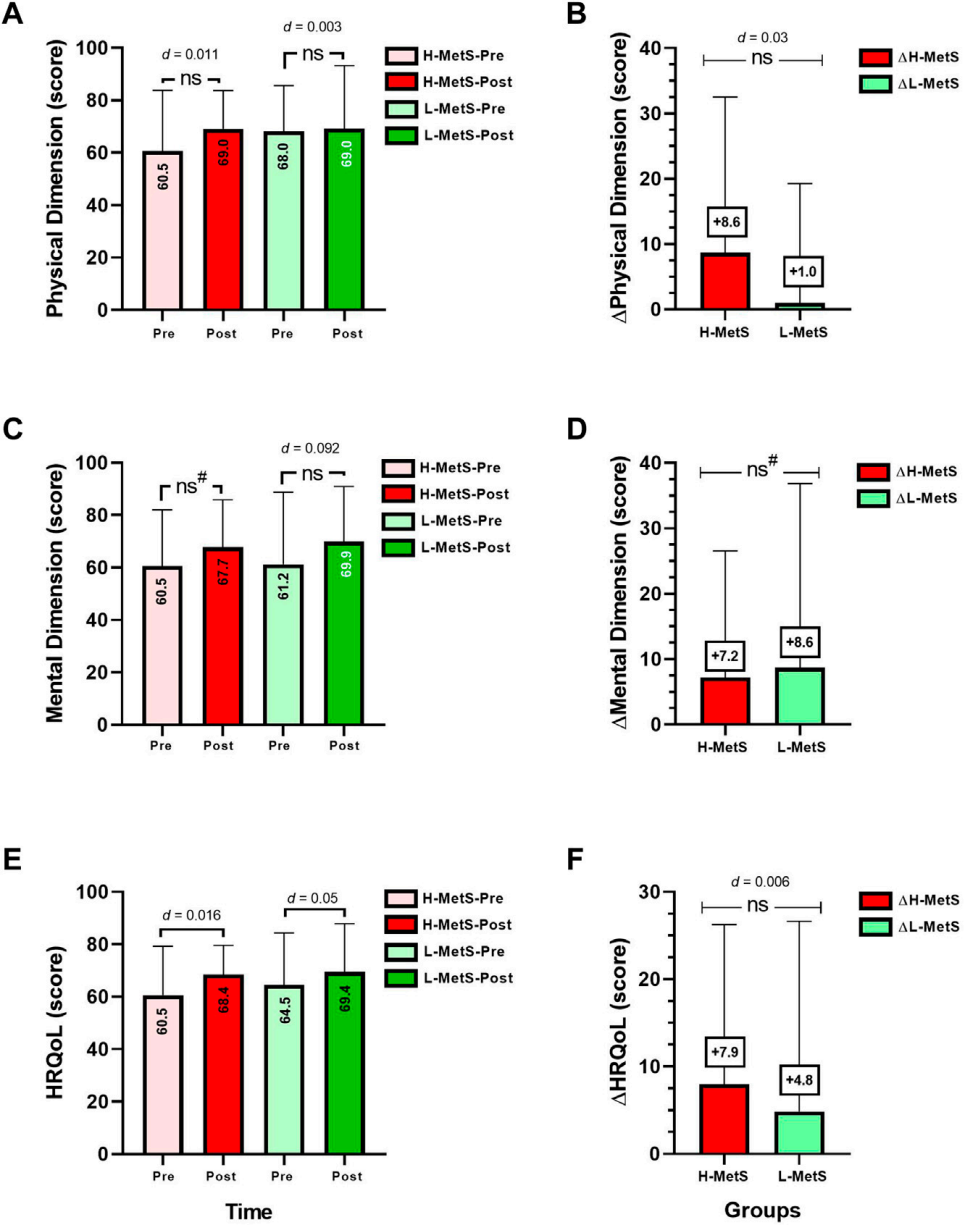
Quality of life dimensions before and after **(A,C,E)**, and delta changes **(B,D,F)** between two groups of a different load of risk factors for metabolic syndrome. Groups are described as H-MetS-Pre, high metabolic syndrome risk factor group at pre test; H-MetS-Post, high metabolic syndrome risk factor group post test; L-MetS-Pre, low-metabolic syndrome risk factor group at pre test; L-MetS-Post, low-metabolic syndrome risk factor group at post test; HRQoL, health-related quality of life scores from the SF-36 instrument. (*d*) Denotes Cohen *d* effect size at *p* < 0.05. (ns) Denotes no significant differences between groups. (^#^) Denotes the comparison analyzed by the Mann–Whitney non-parametric test.

The comparison of HRQoL between H-MetS vs. L-MetS in absolute (Figure 4E) and delta change data (Figure 4F) revealed no significant differences between the groups, in physical (Figures 4A, B) and mental health dimension (Figures 4C, D). However, the interindividual data for HRQoL showed that 29.5 % of the participants, in the H-MetS group, and 45.5 % of the participants, in the L-MetS group, did not elicit improvements (i.e., increases) in the HRQoL score from the instrument applied (Supplementary Material S2).

## Discussion

The objective of the present study was to determine the NR prevalence after 20 weeks of concurrent training on morbidly obese women with a high or a low number of MetS risk factors. The main findings of this study were as follows: 1) it was observed that not all H-MetS participants showed a high NR prevalence in percentage in comparison to those with a lower number of MetS risk factors from the L-MetS group; and 2) 20 weeks of concurrent training promoted clinically beneficial improvements in L-MetS, decreasing ΔWC (−3.8 cm), but particularly in the H-MetS group, ΔSBP (−10.2 mmHg), ΔFPG (−5.8 mg/dL), and ΔHDL-c increased (+4.0 mg/dL), and ΔTg decreased (−8.8 mg/dL), significantly supporting the concurrent training exercise program in delaying the progression of hypertension and type 2 diabetes mellitus, particularly in those morbidly obese patients with a high number of metabolic risk factors (Figure 2).

The literature contains a vast amount of evidence from exercise training intervention in populations with risk factors, where both the effects and the interindividual response have been reported; however, in morbidly obese patients, data are scarce. In terms of the amount of MetS risk factors, previous studies have shown that 8 weeks of concurrent training [24 sessions of RT/3 sets until muscle failure, 1–2-min interval; at 60 %−70 % of one-maximum repetition (1RM); moderate-intensity continuous training (MICT) developed from 5 to 35 min at 65 %–70 % of heart rate at rest] decreased ΔWC (−9.5 cm) (Monteiro-Lago et al., 2019). However, in the present study, we found minor decreases in WC, where we presume that the complex inflammation processes in morbid obesity can alter the normal response to exercise training.

According to the cardiovascular parameters, in the present study, the H-MetS group SBP decreased significantly (142.7 vs. 134.4 mmHg). Previous studies have shown that after 12 weeks of MICT (60 min treadmill) or concurrent training (40 min of MICT on a treadmill 20 min of RT at 60% of 1RM, with 2 min of rest), the participants decreased the 24-h ΔSBP in each protocol (from −8.4 to −7.6 and −8.8 to −7.1 mmHg) to each MICT and the concurrent training group in hypertensive patients (Caminiti et al., 2021), respectively. Other studies on subjects with obesity reported that 12 weeks of concurrent training (60 min per session plus RT three to four sets, six to eight MICT, and RT, using 60–120 s of rest), showed a significant decrease in ΔSBP (−10.81 mmHg) and increased the left ventricular end-diastolic diameter, thus improving cardiac function. Other pieces of evidence reported in women with obesity revealed that HIIT (4 min × 4 min at 85 %–95 % of maximum heart rate, interspersed with 3-min rest periods) and MICT alone (41 min at 65 %–75 % of maximum heart rate) decreased arterial stiffness, and interestingly, HIIT significantly reduced brachial ΔSBP (−6.3) and central ΔSBP (−6.6 mmHg) (De Oliveira et al., 2020), where comparing with the present study, our results show higher ΔSBP reduction (−10.2 mmHg) than previous evidential studies. A part of the mechanisms, by which exercise training decreases blood pressure, is explained by the angiogenesis in skeletal muscle mass (Fernandes et al., 2012), a reduction in peripheral vascular resistance (Correia et al., 2015), a reduction in arterial stiffness (Guimaraes et al., 2010), improvements in the endothelial-mediated vasodilation mechanisms (Ramirez-Jimenez et al., 2017), an increase in production and action of nitric oxide plasma levels (Izadi et al., 2018), and the health status and mode of exercise (Álvarez et al., 2018; De Oliveira et al., 2020). Additional mechanisms explaining why concurrent training decreases blood pressure could include a major baroreflex control (Somers et al., 1991), the shear stress produced by exercise in the arterial wall (Hong et al., 2022) and thus, major arterial distensibility (Kobayashi et al., 2022).

Our results also show other clinical relevance in secondary outcomes. For example, 8 weeks of concurrent training (30 min at 80 % of maximum heart rate plus 2 sets and 10 repetitions at 50 % of the 1RM) significantly reduced ΔTc (−0.76 mg/dL), ΔLDL-c (−1.0 mg/dL), and ΔTg (−0.27 mg/dL) and increased ΔHDL-c (+0.36 mg/dL) in adult subjects (Ghahramanloo et al., 2009). After 16 weeks of concurrent training (40 min MICT and/or RT using a Borg 12-16) and counseling strategy (four educational sessions of a healthy lifestyle), it was reported that the concurrent training group reduced (−9.5 mg/dL) ΔLDL-c in the counseling group but reported an increased ΔHDL-c (+0.90 mg/dL) and reduced ΔFPG (−4.07 mg/dL) in women (Coll-Risco et al., 2018). Concurrent training has a demonstrated capacity for improving MetS risk factors in women, for example, from our previous experience, a recent study of 20 weeks of two concurrent training protocols (HIIT 60 s of a maximum-intensity exercise on bicycle followed by 60–120 s of passive recovery and the intensity established with Borg scale/three out of four RT exercises were included in a similar time of work and rest) reported a decrease in ΔTc (−15.0 mg/dL) and a reduction in ΔTg (−10 mg/dL) (Delgado-Floody et al., 2021).

In terms of the interindividual responses, in other previous literature reports, 9 months of RT, including diet intervention, reported 7.2% NRs to a decrease in WC and 8.6% NRs to a decrease in fat mass percentage (Gremeaux et al., 2012). In other reports of NRs to exercise training, 20 weeks of MICT (30–50 min/session, 3 days/week, 55 %–75 % of the cardiorespiratory fitness) reported 12.2 % NRs to decreased SBP in a sample of ∼1,600 subjects, as was shown by the original authors (Bouchard et al., 2012). In other evidence, following 6 months of MICT (65 %–80 % peak of oxygen uptake, walking/jogging), RT (8–12 repetitions, eight exercises, 70 %–85 % of 1RM, 3 days/week) or concurrent training, there was an NR prevalence of ∼60.9 % for decreasing SBP and ∼59.1 % for decreased DBP (Moker et al., 2014). In the present study, we found an NR prevalence in SBP of 72.7 % (eight subjects) in the H-MetS group and 47.0 % (eight subjects) in the L-MetS group (Figure 4), where it is interesting to note that the non-response could be indirectly influenced by a high degree of disease or inflammation as is the case of the H-MetS group, which showed several significant training-induced change improvements in the MetS outcomes (SBP, FPG, HDL-c, and Tg, Figures 1, 2) but at the same time reported a major prevalence of NRs in the same outcomes as L-MetS peers (Figure 3). Regarding this novel situation of breaking the rule in the sense that usually the higher baseline of the altered/condition leads to more exercise improvements in health outcomes, and thus, minor NR prevalence in percentages, it is necessary to carry out more complex studies in the future.

On the other hand, although we detected that both groups did not improve their quality of life in average terms (HRQoL score of the SF-36 instrument) and no significant differences were detected between the groups (Figure 4E, F), interindividual data revealed that 29.5% of the H-MetS group and 45.5% of the L-MetS group showed no changes or improvements in the HRQoL score (Supplementary Material S2). From these results, we presume that considering we revealed a relevant percentage of NRs in each H-MetS and L-MetS group, there is a clear presumption that as these were not improved/or worsened in the same cases, participants should not have improved their quality of life. Our results are in coherence with those of a previous study conducted on adults for 21 weeks of MICT (cycling, the intensity was based on the MICT, and another regime at anaerobic thresholds/30–90 min) and concurrent training (MICT + RT, three to four training sets per session/1–3-min rest intervals between sets) in which it was shown that both exercise modalities improved several physical, mental, and social dimensions of the HRQoL (Sillanpää et al., 2012).

The present study revealed several clinical implications, for example, from our ∆SBP results (H-MetS ∆SBP −10.2 ± 11.4; L-MetS −3.2 mmHg), a 2 mmHg reduction in SBP decreases mortality from cerebral vascular accidents by 10 % and cardiovascular diseases by 7 % (Collaboration, 2002; Guimaraes et al., 2010). Additionally, a reduction of 2 mmHg in SBP reported improvements (i.e., decreases) in the functional/structural marker of the endothelial function pulse wave velocity by −0.54 m/s (Guimaraes et al., 2010), and more recently, a reduction of −3.2 mmHg in SBP after concurrent training was related to an increase in flow-mediated dilation (+6.8%), which also led to clinical benefits (Pedralli et al., 2020).

Our study is not without its limitations: 1) we did not include strict control groups; however, the L-MetS group was used as a comparator; 2) we did not analyze the differences between the H-MetS vs. L-MetS groups’ testing clinically significant standard deviation differences; however, we used the widely known TE × 2 measurements from the original authors (Bouchard et al., 2012); 3) we did not monitor the diet strictly; however, there was a reminder each week to participants about maintaining their baseline patterns; and finally, 4) our sample size was not large enough to extrapolate; however, under an experimental clinical study, the sample size was calculated statistically and *n* = 10 was a frequent sample size used in exercise training interventions. As for strengths, we have contributed to the limited scientific evidence on the rate of Rs and NRs in morbidly obese women, and additionally, we reported the information on several secondary outcomes and their responses after a concurrent training exercise in this poorly studied population and their cardiometabolic risk.

In conclusion, 20 weeks of concurrent training promotes greater beneficial effects in morbidly obese patients with higher MetS risk factors. However, the NR prevalence for improving MetS outcomes was significantly superior in the more diseased groups in SBP, FPG, and HDL-c, independent of their major training-induced effects.

## Data Availability

The original contributions presented in the study are included in the article/Supplementary Materials; further inquiries can be directed to the corresponding author.

## Supplementary material

SUPPLEMENTARY MATERIAL S1 Study design.

SUPPLEMENTARY MATERIAL S2 Health-related quality of life changes after 20 weeks of concurrent training in women with morbid obesity.

## Abbreviations

MetS, metabolic syndrome; WC, waist circumference; Tg, triglycerides; HDL-c, high-density lipoprotein cholesterol; Tc, total cholesterol; Rs, responders; NRs, non-responders; H-MetS, high load of metabolic syndrome risk factors group; L-MetS, low load of metabolic syndrome risk factors group; BMI, body mass index; FPG, fasting plasma glucose; LDL-c, low-density lipoprotein cholesterol; SBP, systolic blood pressure; DBP, diastolic blood pressure; HRQoL, health-related quality of life; RT, resistance training; HIIT, high-intensity interval training.
