# Supplementary figures and images for: Concurrent training and interindividual response in women with a high number of metabolic syndrome risk factors
Source: Front Physiol. 2022 Sep 23;13:934038. doi: 10.3389/fphys.2022.934038 (PMC9546759; doi:10.3389/fphys.2022.934038)

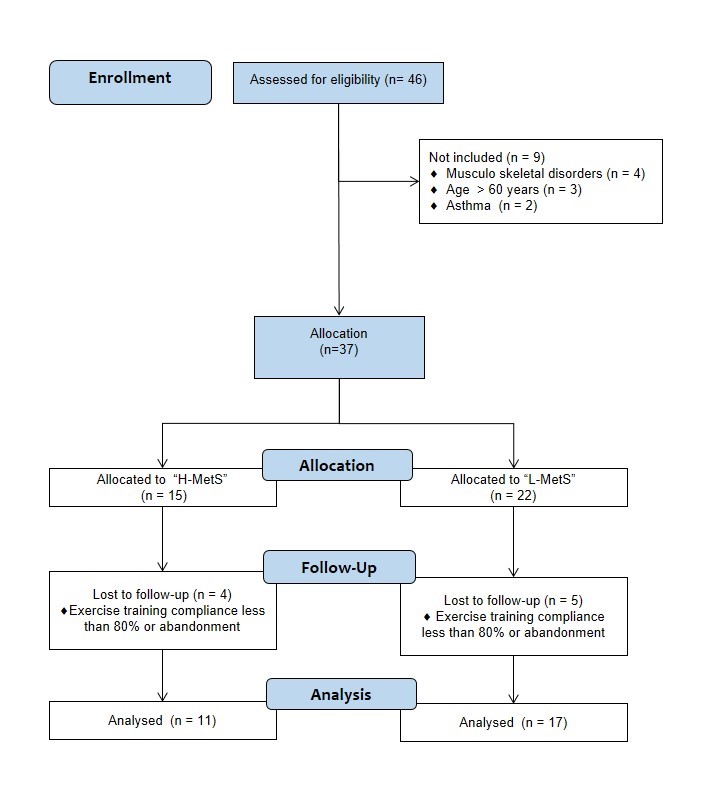

Supplement: Supplementary file 1 [file Image1.JPEG]

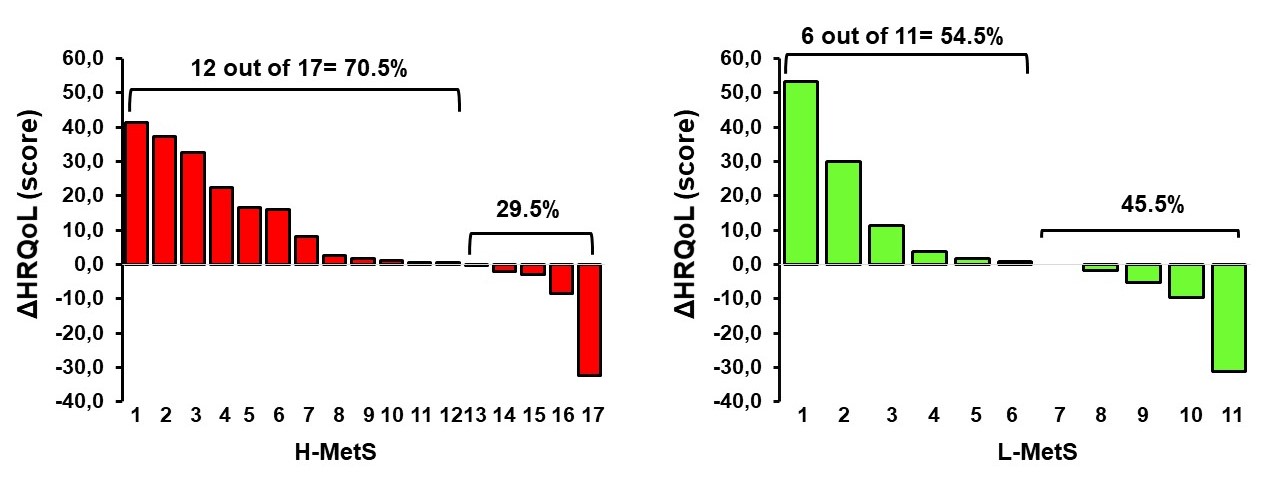

Supplement: Supplementary file 2 [file Image2.JPEG]
